# Mouse *Panx1* Is Dispensable for Hearing Acquisition and Auditory Function

**DOI:** 10.3389/fnmol.2017.00379

**Published:** 2017-11-28

**Authors:** Veronica Zorzi, Fabiola Paciello, Gaia Ziraldo, Chiara Peres, Flavia Mazzarda, Chiara Nardin, Miriam Pasquini, Francesco Chiani, Marcello Raspa, Ferdinando Scavizzi, Andrea Carrer, Giulia Crispino, Catalin D. Ciubotaru, Hannah Monyer, Anna R. Fetoni, Anna M. Salvatore, Fabio Mammano

**Affiliations:** ^1^CNR Institute of Cell Biology and Neurobiology, Monterotondo, Italy; ^2^School of Medicine, Institute of Otolaryngology, Catholic University, Rome, Italy; ^3^Department of Science, Roma Tre University, Rome, Italy; ^4^Department of Biology and Biotechnology Charles Darwin, Sapienza University of Rome, Rome, Italy; ^5^Department of Physics and Astronomy G. Galilei, University of Padua, Padua, Italy; ^6^CNR Institute of Neuroscience, Padua Section, Padua, Italy; ^7^Department of Clinical Neurobiology, Deutches Krebforschungzentrum, University of Heidelberg, Heidelberg, Germany; ^8^Shanghai Institute for Advanced Immunochemical Studies, ShanghaiTech University, Shanghai, China

**Keywords:** cochlea, pannexins, connexins, hair cells, non-sensory cells, auditory brainstem responses, distortion product otoacoustic emissions

## Abstract

Panx1 forms plasma membrane channels in brain and several other organs, including the inner ear. Biophysical properties, activation mechanisms and modulators of Panx1 channels have been characterized in detail, however the impact of *Panx1* on auditory function is unclear due to conflicts in published results. To address this issue, hearing performance and cochlear function of the *Panx1*−/− mouse strain, the first with a reported global ablation of *Panx1*, were scrutinized. Male and female homozygous (*Panx1*−/−), hemizygous (*Panx1*+/−) and their wild type (WT) siblings (*Panx1*+/+) were used for this study. Successful ablation of *Panx1* was confirmed by RT-PCR and Western immunoblotting in the cochlea and brain of *Panx1*−/− mice. Furthermore, a previously validated Panx1-selective antibody revealed strong immunoreactivity in WT but not in *Panx1*−/− cochleae. Hearing sensitivity, outer hair cell-based “cochlear amplifier” and cochlear nerve function, analyzed by auditory brainstem response (ABR) and distortion product otoacoustic emission (DPOAE) recordings, were normal in *Panx1*+/− and *Panx1*−/− mice. In addition, we determined that global deletion of *Panx1* impacts neither on connexin expression, nor on gap-junction coupling in the developing organ of Corti. Finally, spontaneous intercellular Ca^2+^ signal (ICS) activity in organotypic cochlear cultures, which is key to postnatal development of the organ of Corti and essential for hearing acquisition, was not affected by *Panx1* ablation. Therefore, our results provide strong evidence that, in mice, *Panx1* is dispensable for hearing acquisition and auditory function.

## Introduction

*Panx1* is the most thoroughly characterized member of the pannexin gene family (Panchin et al., 2000; Bruzzone et al., 2003; Baranova et al., 2004) encoding Panx1, Panx2 and Panx3 proteins^1^ that form plasma membrane channels known as pannexons (Sosinsky et al., 2011). Biophysical properties, activation mechanisms and modulators of Panx1 channels have been extensively reviewed (Dahl et al., 2013; Penuela et al., 2013; Dahl and Muller, 2014; Patel et al., 2014; Esseltine and Laird, 2016).

Quantification of Panx1 mRNA levels by quantitative real-time polymerase chain reaction (QPCR) in mouse central and peripheral nervous system, and various organs, revealed highest values in trigeminal ganglia > bladder > spleen, followed at distance by hippocampus > cortex ~ calvaria > heart > cerebellum, with lowest levels in kidney and spleen (Hanstein et al., 2013). Furthermore, Panx1 has been localized in eye (Ray et al., 2005; Kurtenbach et al., 2014), taste buds (Huang et al., 2007), olfactory system (Zhang et al., 2012) and inner ear (Tang et al., 2008; Wang et al., 2009; Zhao, 2016). As for the latter, the impact of *Panx1* on auditory function is unclear due to recent publication of conflicting results.

The first *Panx1* knockout mice (*Panx1*^tm1Mony^, International strain designation B6;129-*Panx1*^tm1.1Fama/Cnrm^; EMMA ID:11476)^2^, hereafter referred to briefly as *Panx1*−/−, were reported in Anselmi et al. (2008). A subsequent study used *in situ* hybridization and western blot in brain extracts to confirm successful *Panx1* deletion in these mice (Bargiotas et al., 2011). The same study determined that the following antibodies were not specific: goat anti-Panx1, sc-49695 (Santa Cruz); chicken anti-Panx1, no. ANT0027 (Diatheva); chicken anti-human Panx1, #4515, provided by G. Dahl (University of Miami School of Medicine); rabbit anti-Panx1, provided by G. Zoidl (Bochum, Germany). *Panx1*−/− mice displayed altered electroretinograms in response to light flashes (Kranz et al., 2013), whereas no auditory phenotype was detected by auditory brainstem response (ABR) analysis (Anselmi et al., 2008,^3^).

Other researchers used mice with loxP sites flanking exon 2 of *Panx1*, obtained from Genentech. Deletion of exon 2 (which introduces a frameshit and premature stop codon), was accomplished by breeding to C57BL/6-*Gt(ROSA)26Sor*^tm16(Cre) Arte^ (TaconicArtemis), a ubiquitously active Cre deleter line (Otto et al., 2009). Offspring were backcrossed several times to C57BL/6 mice, to breed out the Cre recombinase, resulting in a knockout strain henceforth referred to as Genentech-*Panx1*−/−. Successful ablation of *Panx1* was confirmed in several organs on these mice (muscle, lung, liver, kidney, tail, brain, heart, thymus, spleen and skin tissues; Qu et al., 2011; Cone et al., 2013; Penuela et al., 2014) as well as in the cochlea (Abitbol et al., 2016). Genentech**-***Panx1*−/− mice exhibited neither reduced hearing sensitivity nor cochlear nerve defects; furthermore, susceptibility to noise-induced hearing loss in these mice was indistinguishable from that of their wild type (WT) controls (Abitbol et al., 2016).

A strain carrying the *Panx1^tm1a(KOMP)Wtsi^* allele was generated by the Knock Out Mouse Project (KOMP^4^) using the multipurpose tm1a knockout-first promoter-driven selection cassette, which has been adopted also by other major mouse knockout programs such as EUCOMM (EUropean Conditional Mouse Mutagenesis Program^5^; Collins et al., 2007). The versatile tm1a allele contains an IRES:lacZ trapping cassette and a floxed promoter-driven *neo* cassette inserted into the intron of a gene, disrupting gene function^6^. However, mice homozygous for the *Panx1*^tm1a(KOMP)Wtsi^ knockout-first promoter-driven allele were found to express about 30% residual *Panx1* mRNA in all organs examined, leading to the conclusion that *Panx1*^tm1a(KOMP)Wtsi^ is a hypomorphic allele (Hanstein et al., 2013). Hypomorphism has been reported also for other knockout-first alleles (Shpargel et al., 2012; Ryder et al., 2014).

Aiming to achieve *Panx1* conditional deletion in the cochlea, Chen et al. (2015) crossed the hypomorphic *Panx1*^tm1a(KOMP)Wtsi^ mice with *Foxg1*-Cre mice (Hébert and McConnell, 2000; Bredenkamp et al., 2007), and identified homozygous *Foxg1*^Cre^:*Panx1*^f/f^ mice as conditional *Panx1* knockout mice (henceforth referred to as *Foxg1*-*cPanx1*−/−). Intense immunofluorescence labeling with the non-specific chicken anti-human Panx1 antibody #4515 was detected in the spiral limbus and organ of Corti, but not in the lateral wall of *Foxg1*-*cPanx1*−/− mice, which had significantly elevated hearing thresholds at all frequencies, reduced endocochlear potential and reduced cochlear microphonics (Chen et al., 2015).

Zhao et al. (2015) crossed *Panx1^tm1a(KOMP)Wtsi^* mice with *Paired box* 2 Cre (*Pax2*-Cre) mice (Tian et al., 2006; Ohyama, 2009) to generate yet another strain of *Panx1* conditional knockout in the cochlea (henceforth referred to as *Pax2*-*cPanx1*−/−). ABR thresholds of *Pax2*-*cPanx1*−/− mice were 40 dB sound pressure level (SPL) greater than in WT littermates at P80 (where P0 is day of birth). Likewise, DPOAEs were significantly decreased in *Pax2*-*cPanx1*−/− mice compared to WT (Zhao et al., 2015) at P50.

In the light of these contrasting results, the goal of the present study was to clarify the role of *Panx1* in hearing. To this end, we re-evaluated hearing performance and cochlear function of *Panx1*−/− mice using *in vivo* electrophysiology, plus a variety of biochemical and biophysical assays.

## Materials and Methods

### Ethics Statement

Animal work was performed in accordance with a protocol approved by the Italian Ministry of Health (Authorization n.1005/2016-PR, date 21/10/2016, DGSAF Prot. No. 002451-P-25/10/2016 and No. 0001276-P-19/01/2016).

### Animals and Genotyping

Animals were bread and genotyped in the CNR Monterotondo node of the European Mouse Mutant Archive (EMMA; del Hierro et al., 2016), an ESFRI/INFRAFRONTIER Distributed Research Infrastructure^7^.

Mice tested were P5 pups and adults (1–3 months of age). Male and female homozygous (*Panx1*−/−), hemizygous (*Panx1*+/−) and their WT siblings (*Panx1*+/+) were used for this study. The background strains of these mice was C57BL/6N.

*Panx1* mice were genotyped according to published protocols by standard PCR on extracted mouse tail tips (Anselmi et al., 2008; Bargiotas et al., 2011) using the following primers:

- *Panx1* f: 5′-GGAAAGTCAACAGAGGTACCC-3′
- *Panx1* r: 5′-CTTGGCCACGGAGTATGTGTT-3′
- LacZ: 5′-GTCCCTCTCACCACTTTTCTTACC-3′

The *Panx1*+/+ allele was targeted by the above f and r primers and identified by a 330 bp band, whereas *Panx1*−/− was targeted by primers *Panx1* f and LacZ, and was identified by a 630 bp band. *Panx1*+/− was identified by the simultaneous presence of a 330 bp and a 630 bp band.

For some internal control experiments, mice with ubiquitous deletion of connexin 30 (*Cx30*−/−; International strain name B6.129P2-*Gjb6*^tm1Kwi/Cnrm^; EMMA ID: 00323; MGI ID: 2447863; Teubner et al., 2003; Cohen-Salmon et al., 2007; Schütz et al., 2010; Johnson et al., 2017) were also used. The background strains of these mice was C57BL/6J. *Cx30*−/− mice were genotyped as indicated in the protocol provided by INFRAFRONTIER:

- Cx30 f: 5′-GGTACCTTCTACTAATTAGCTTGG-3′
- Cx30 r: 5′-AGGTGGTACCCATTGTAGAGGAAG-3′
- LacZ: 5′-AGCGAGTAACAACCCGTCGGATTC-3′

*Cx30*+/+ mice were identified by a 544 bp band, whereas *Cx30*−/− mice were identified by a 460 bp band.

### RNA Extraction and RT-PCR

Following euthanasia, cochleae and brains were dissected from adult and P5 *Panx1*+/+ and *Panx1*−/− mice and flash-frozen in liquid nitrogen. Total RNA was extracted with RNeasy mini kit (Qiagen, Cat. No. 74106) and retrotranscribed with oligo–dT 12–18 primers (ThermoFisher, Cat. No. 18418012) using Omniscript RT kit (Qiagen, Cat. No. 205111). Subsequently RT-PCR was performed using PCR BIO HS Taq Mix Red (PB10.23–02). The PCR profile was: 95°C for 5 min, 94°C for 15 s, 62°C for 30 s, 72°C for 15 s and 72°C for 2 min for 34 cycles. Primers used were:

- *Panx1* ex3 f: 5′-ACACCTCTGCTCAGACCTGAA-3′
- *Panx1* ex4 r: 5′-TGCACAGAAACTCGCGTCCGAGA-3′
- *GAPDH* f: 5′-ATGTGTCCGTCGTGGATCTGAC-3′
- *GAPDH* r: 5′-AGACAACCTGGTCCTCAGTGTAG-3′

**Figure 1:**
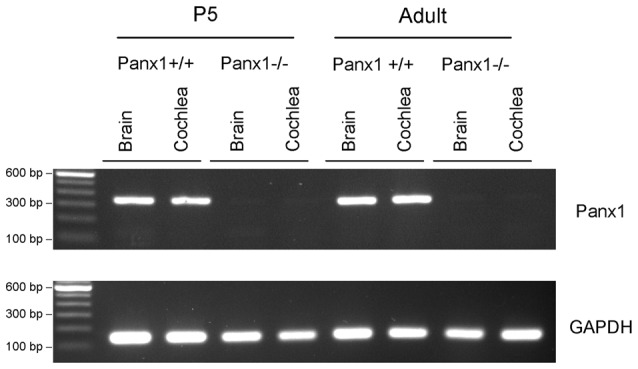
RT-PCR analysis of brain and cochlea Panx1 and GAPDH mRNA transcription in wild type (*Panx1*+/+) and *Panx1*−/− mice.

The amplified products were run on a 1, 5% agarose gel with SYBR Safe (ThermoFisher, Cat. No. S33102) for visualization of the *Panx1* band (336 bp) and the *GAPDH* band (132 bp) (Figure 1).

### QPCR

QPCR was performed on cDNA to amplify *Cx26* and *Cx30* and was normalized to *GAPDH*. Gene expression relative to *GAPDH* was estimated according to a published method (Pfaffl, 2001). Amplification was carried out using Power SYBR Green (Applied Biosystems, Cat. No. 4367659) on the ABI 7700 sequence detection system equipped with ABI Prism 7700 SDS software (Applied Biosystems) through the following amplification cycles: 50°C for 2 min, 95°C for 10 min, 95°C for 15 min, 60°C for 1 min (40 cycles). Primers used are as follows:

- *Cx26* f: 5′-TCACAGAGCTGTGCTATTTG-3′
- *Cx26* r: 5′-ACTGGTCTTTTGGACTTTCC-3′
- *Cx30* f: 5′-GGCCGAGTTGTGTTACCTGCT-3′
- *Cx30* r: 5′-TCTCTTTCAGGGCATGGTTGG-3′
- *GAPDH* f: 5′-ATGTGTCCGTCGTGGATCTGAC-3′
- *GAPDH* r: 5′-AGACAACCTGGTCCTCAGTGTAG-3′

### Western Immunoblotting

Total proteins were extracted from brains and cochleae of P5 and adult *Panx1*+/+ (*n* = 4) and *Panx1*−/− mice (*n* = 4). Tissues were dissected, collected on liquid nitrogen, stored at −80°C and homogenized by using ice cold RIPA buffer (Pierce; 50 mM Tris, 150 mM NaCl, 1 mM EDTA, 1% DOC, 1% Triton X-100, 0.1% SDS, and 1× protease, phosphatase-1, and phosphatase-2 inhibitor cocktails [Sigma]). The lysate was sonicated three times at 10 Hz (Hielscher, Ultrasound Technology UP50H/UP100H), centrifuged (13,000 rpm, 15 min, 4°C), and a 5 μl aliquot of the supernatant was assayed to determine the protein concentration (microBCA kit, Pierce). SDS-PAGE reducing sample buffer was added to the supernatant, and samples were heated to 95°C for 5 min. Protein lysates (70 μg) were loaded onto 12% Tris-glycine polyacrylamide gels for electrophoretic separation. ColorburstTM Electrophoresis markers (Sigma) were used as molecular mass standards. Proteins were then transferred onto nitrocellulose membranes at 100 V for 2 h at 4°C in transfer buffer containing 25 mM Tris, 192 mM glycine, 0.1% SDS and 20% methanol. Membranes were incubated for 1 h with blocking buffer (5% skim milk in TBST), and then incubated overnight at 4°C with primary antibodies directed against Panx1 (1 mg/ml, ThermoFisher, Cat. No. 487900), Cx26 (1 mg/ml, ThermoFisher, Cat. No. 512800), Cx30 (1 mg/ml, ThermoFisher, Cat. No. 712200) and GAPDH (1:2500, Abcam). After three 10 min rinses in TBST, membranes were incubated for 1 h at room temperature (22–25°C) with HRP-conjugated secondary antibodies (Cell Signaling, 1:2500). The membranes were then washed, and the bands were visualized with an enhanced chemiluminescence detection kit (GE Healthcare, UK). Protein expression was evaluated and documented by using a UVItec Cambridge Alliance system.

### Immunohistochemistry and Confocal Imaging

Cochleae were extracted from P5 and adult mice and processed as previously described (Crispino et al., 2011, 2017). Briefly, samples were fixed in 4% paraformaldehyde and decalcified in ethylenediaminetetraacetic acid (EDTA, 0.3 M). Specimens were included in 3% agarose dissolved in PBS and cut in 100 μm thickness steps using a vibratome (VT 1000 S, Leica). Tissue slices were permeabilized with 0.1% Triton X-100, dissolved in bovine serum albumin 2% solution. Panx1 was immunolabeled by overnight incubation at 4°C with a chicken anti-Panx1 specific antibody (1:500; extracellular loop epitope: VQQKSSLQSES, AvesLab #6358; Hanstein et al., 2013) provided by Prof. Eliana Scemes. Cx30 and Cx26 were immunolabeled by overnight incubation at 4°C respectively with a rabbit polyclonal Cx30 and a mouse monoclonal Cx26 selective antibody (10 μg/ml, ThermoFisher, Cat. No. 712200 for Cx30; Cat. No. 335800 for Cx26). Secondary antibodies (10 μg/ml) were: Alexa Fluor^®^ 488 goat anti-chicken IgY (H+L), ThermoFisher, Cat. No. A11039; Alexa Fluor^®^ 488 goat anti-rabbit IgG, ThermoFisher, Cat. No. A11008; Alexa Fluor^®^ 488 goat anti-mouse IgG, ThermoFisher, Cat. No. A11029), applied at room temperature (22–25°C). F–Actin was stained by incubation with AlexaFluor 568 phalloidin (1 U/ml, ThermoFisher, Cat. No. A12380), and nuclei were stained with 4′,6′diamidino–2′phenylindole (DAPI, ThermoFisher, Cat. No. D1306; 1:200). The same immunostaining procedure was used also for organotypic cultures from P5 pups. All samples were mounted onto glass slides with a mounting medium (FluorSaveTM Reagent, Merk, Cat. No. 345789) and analyzed using a confocal microscope (TCS SP5, Leica) equipped with an oil–immersion objective (40× HCX PL APO 1.25 N.A., Leica).

### ABR and DPOAE Measurement

Auditory function was assessed in a sound-attenuating enclosure (ETS-Lindgren SD Test Enclosure, MDL Technologies Limited, Hitchin, UK) using an ABR Workstation (Tucker-Davis Technologies, Inc., Alachua, FL, USA) comprising: Z-Series 3-DSP Bioacoustic System w/Attenuators and Optic fiber; Medusa 4-Channel Pre-Amp/Digitizer; Medusa 4-Channel Low Imped. Headstage; MF1-M Multi Field Magnetic Speakers—Mono; AEP/OAE Software for RZ6; Experiment Control Workstation. Sound levels were calibrated using a $\frac{1}{4}$ inch Free Field Measure Calibration Microphone Kit (Model 480C02; PCB).

Mice were anesthetized with intraperitoneal injections of ketamine (70 mg/g for males, 100 mg/g for females) and medetomidine (1 mg/g). The depth of anesthesia was periodically verified by the lack of foot-pinch response. Body temperature was maintained at 37°C using a heating pad under feedback control. Corneal drying was prevented by application of ophthalmic gel to the eyes of the animals.

For ABR recordings (Scimemi et al., 2014), acoustic stimuli consisted of clicks (100 μs duration) and tone bursts (1 ms rise–fall time with 3 ms plateau) of 4, 8, 16, 24 and 32 kHz, and were delivered in the free field using a MF1-M speaker. Bioelectrical potentials were collected with gauge 27, 13 mm needle electrodes (Cat. No. S83018-R9, Rochester) inserted subdermally at the vertex (active), ventrolateral to the left ear (reference) and above the tail (ground). Potentials were amplified, filtered (0.3–3 kHz) and averaged over 512 presentations of the same stimulus. Hearing threshold levels were determined offline as the SPL at which a Wave II peak, could be visually identified above the noise floor (0.1 μV).

Otoacoustic emissions (Kemp, 1978) were evoked using a pair of equal intensity primary tones, *f*_1_ = 14,544 Hz, and *f*_2_ = 17,440 kHz delivered at intensities ranging from 20 to 80 dB SPL in 10 dB SPL increments. Each primary tone (20.97 ms duration, 47/s) was emitted by a separate MF1-M speaker, configured for closed field stimulation, and delivered to the mouse ear via a small tube as prescribed by the manufacturer. The cubic distortion product 2*f*_1_ – *f*_2_ = 11,648 kHz, was detected using a small microphone (ER10B+ Low Noise Probe and Microphone, Etymotic Research, IL, USA) coupled to the ear canal.

### Preparation of Cochlea Organotypic Cultures

Cochleae from P5 mouse pups were quickly dissected in ice-cold Hepes buffered (pH 7.2) HBSS (ThermoFisher, Cat. No. 14025050), placed onto 12 mm glass coverslips coated with Cell-Tak (Biocoat, Cat. No. 354240) and incubated overnight at 37°C in DMEM/F12 (ThermoFisher, Cat. No. 11320-074) supplemented with 5% FBS (ThermoFisher, Cat. No. 10270-106) and 100 μg/ml ampicillin (Sigma-Aldrich, Cat. No. A0166).

### Dye Transfer Assays in Cochlear Organotypic Cultures

To visualize gap junction coupling among non-sensory cells of the lesser epithelial ridge (LER), we performed dye-transfer assays using the fluorescent tracer Lucifer Yellow (LY, CH Lithium Salt, Thermofisher, #L12926) for microinjection. Cochlear cultures were transferred on the stage of a spinning disk confocal microscope (DSU, Olympus) and perfused for 5 min at 1 ml/min with EXM, an extracellular solution containing (in mM): NaCl 135, KCl 5.8, CaCl2 1.3, NaH_2_PO_4_ 0.7, MgCl_2_ 0.9, Hepes−NaOH 10, d−glucose 6, pyruvate 2, amino acids and vitamins (pH 7.48, 307 mOsm). For dye delivery, patch pipettes were fabricated from glass capillaries (G85150T-4, Harvard Apparatus, Edenbridge, UK) using a double stage vertical puller (PP-830, Narishige) and were filled with LY dissolved at 220 μM (final concentration) in a 320 mOsm intracellular solution containing (in mM): KCl 134, NaCl 4, MgCl_2_ 1, HEPES 20, EGTA 10 (adjusted to pH 7.3 with KOH) and filtered through 0.22 μm pores (Millipore). Pipette resistances were 3–4 MOhm when immersed in the bath. One cell (donor) was patch clamped and maintained in the cell-attach configuration for a few seconds to establish a baseline. The patch of membrane under the pipette sealed to the donor cell was subsequently ruptured, allowing the LY to fill the cell, while leaving the seal intact (whole cell recording conditions). The cell was held at its zero current level using the current clamp configuration of the patch clamp amplifier (Axopatch 200B, Molecular devices). LY diffusion among (first order) cells adjacent to the injected cell was monitored over time by acquiring fluorescence images at a rate of 1 frame per second with a typical exposure time of 70 ms. Fluorescence images were displayed as (F − F_bck_)/(F_max_ − F_bck_), where F_max_ is the maximal value reached in the injected cell at the end of the recordin interval and F_bck_ is autofluorescence. Experiments were performed at room temperature (22–25°C).

### Multiphoton Microscopy and Ca^2+^ Imaging in Cochlear Organotypic Cultures

To record spontaneous intercellular Ca^2+^ signal (ICS) activity in nonsensory cells of the mouse cochlea, organotypic cultures of sensory epithelium were incubated for 45 min at 37°C in DMEM/F12 supplemented with the acetoxymethyl (AM) ester of the selective Ca^2+^ sensor Fluo-Forte (16 μM, Enzo Life Science, #ENZ-52014). The incubation medium contains also pluronic F-127 (0.1% w/v, Sigma-Aldrich, #P2443) and sulfinpyrazone (250 μM, Sigma-Aldrich, #S9509) to prevent dye sequestration and secretion. Cultures were then transferred to an upright microscope stage (see below) and continually perfused with EXM (see above) for 15 min at 1 ml/min in a dark environment at 25°C to allow for dye de-esterification. All subsequent imaging experiments were also performed at 25°C.

To record Ca^2+^ signals, we used a two-photon microscope (Bergamo II, Thorlabs) equipped with a resonant scanner and coupled with a mode-locked Ti:Sapphire pulsed laser (Chameleon-Ultra II, Coherent). Fluo-Forte was excited at 940 nm by focusing the Ti:Sapphire beam onto the sample through a water-immersion objective (XLPlan N, 25× 1.05 NA, Olympus). Average power at the sample was ~20 mW. The fluorescence signal, collected by the same objective, was reflected towards the detection arm of the microscope by the 705 nm primary dichroic mirror of the microscope (Semrock, FF705-Di01), placed at 45° above the objective. After traversing a 680 nm short pass filter (FF01-680/SP-25, Semrock) and 495 nm dichroic beam-splitter (T495lpxru, Chroma Technology), the Fluo-Forte signal was selected in the range 435–485 nm by a single band-pass filter (ET460/50m-2p, Chroma Technology) placed in front of a non-descanned GaAsP detector (H7422–50, Hamamatsu). Mechanical ultra-fast shutters were used to limit light exposure to the bear minimum required for image acquisition.

Sequences of 512 × 512 pixels frames were acquired, averaged in lots of nine and presented at a final rate of 5 per second. Illumination intensity, frame average, frame rate and the number of pixels in each frame were adjusted so as to minimize photobleaching and phototoxicity, while achieving sufficient signal to noise ratio and temporal resolution. Image sequences were acquired using ThorImage LS 3.1 software (Thorlabs).

Ca^2+^ signals were quantified as pixel-by-pixel relative changes of fluorescence emission intensity, i.e., Δ*F*(*t*)/*F*_0_ where *t* is time, *F(t)* is fluorescence at time *t*, *F*_0_ is the fluorescence at the onset of the recording and Δ*F*(*t*) = *F*(*t*) − *F*_0_. All data were processed off-line and presented using Vimmaging (F. Mammano and C. Ciubutaru, VIMM, Padova, Italy), a custom-made software routine developed under MATLAB™ environment (The MathWorks Inc., Natick, MA, USA).

## Results

### Panx1 Is Absent in the Cochlea of *Panx1*−/− Mice

**Figure 2:**
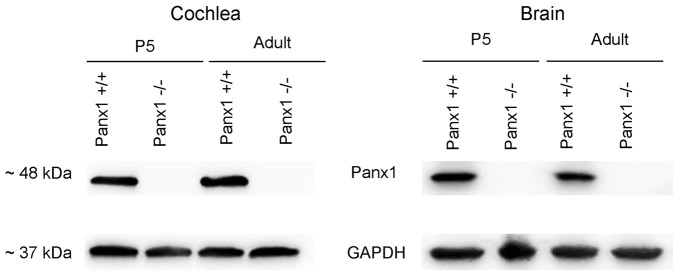
Western blot with a Panx1-selective antibody in cochlea and brain.

Generation and genotyping of *Panx1*−/− mice were previously described (Anselmi et al., 2008; Bargiotas et al., 2011). Here we report an additional data set based on Panx1 expression analyses by RT-PCT and Western immunoblotting. Panx1 mRNA transcript expression was detected in the cochlea and brain of WT mice at both P5 and adult stage, but not in *Panx1*−/− mice (Figure 1). Consistent with these results, Western blots failed to reveal Panx1 expression in the cochlea of *Panx1*−/− mice at both P5 and in the adult stage, whereas bands with the correct molecular weight (~48 kDa) were present in *Panx1*+/+ (i.e., WT) extracts (Figure 2).

**Figure 3:**
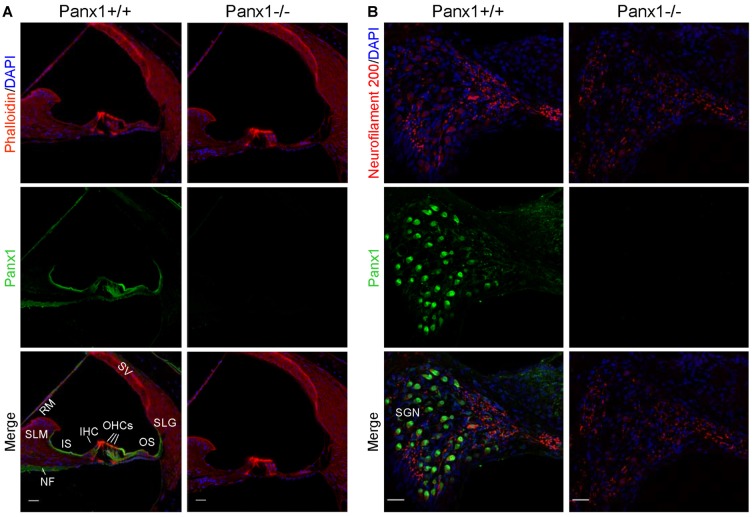
Immunofluorescence with a validated Panx1-selective antibody in cochlear midmodiolar sections. **(A)** Representative transversal sections of scala media. Shown are maximal projection rendering of three consecutive confocal optical sections taken at 0.5 μm intervals in the apical cochlear turn; actin filaments were stained with phalloidin (red) and nuclei with DAPI (blue); Panx1 expression was detected with the AvesLab #6358 selective antibody (green). IHC, inner hair cells; OHCs, outer hair cells; RM, Reissner’s membrane; SV, stria vascularis; SLM, spiral limbus; SLG, spiral ligament; IS, inner sulcus; OS, outer sulcus; NF, nerve fibers. Gamma filters were applied to both red (γ = 0.40) and green (γ = 0.55) channels. Scale bars: 25 μm. **(B)** Representative transversal sections of spiral ganglion. Maximal projection rendering of 10 consecutive confocal optical sections taken at 1.5 μm intervals in the medial cochlear turn; nerve fibers were stained with a neurofilament 200 selective antibody (red). SGN, spiral ganglion neurons. Gamma filters were applied to both red (γ = 0.55) and green (γ = 0.9) channels. Scale bars: 25 μm.

Using a previously validated antibody that targets an extracellular epitope of the Panx1 protein (AvesLab #6358; Hanstein et al., 2013), we detected strong immunoreactivity in epithelial cells lining the endolymphatic surface of the sensory epithelium (inner sulcus, outer sulcus), supporting and epithelial cells of the organ of Corti, Reissner’s membrane, and spiral ganglion neurons of WT mice. Weak immunostaining was also detected in the spiral limbus and spiral ligament of these mice, whereas the AvesLab #6358 antibody failed to label cochlear tissue from *Panx1*−/− mice (Figure 3). Altogether these results confirm successful ablation of *Panx1* in the (brain and) cochlea of *Panx1*−/− mice.

### ABRs and DPOAEs in *Panx1*−/− Mice Are Indistinguishable from WT Controls

Next, we sought to corroborate and extend the results obtained by Anselmi et al. (2008) by analyzing in greater detail the hearing performance of *Panx1*−/− mice. In humans and mice alike, sound-evoked ABR potentials appear as a series of consecutive relative maxima (peaks), termed Waves and labeled with Roman numerals, which arise from the synchronous short-latency synaptic activity of successive nuclei along the peripheral afferent auditory neural pathway (Zheng et al., 1999; Legatt, 2002; Zhou et al., 2006). The first peak (Wave I) arises from the cochlea and/or compound action potential of the auditory nerve ~1 ms after the stimulus onset (latency). Waves from II to V originate from cochlear nuclei, contralateral superior olivary complex, lateral lemniscus and contralateral lateral inferior colliculus. For reference, Table I of Scimemi et al. (2014) presents means and standard deviations (SD) of latency and amplitude values of ABR peaks I–V for C57BL/6 mice.

**Figure 4:**
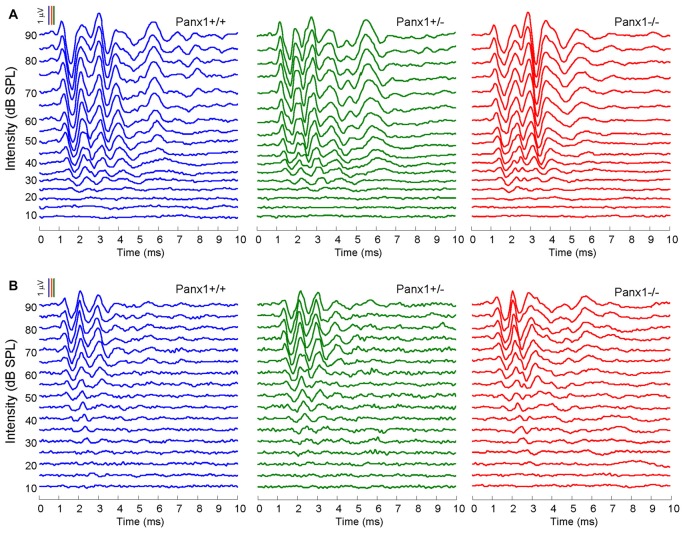
*In vivo* electrophysiological recordings. **(A,B)** Representative recordings of auditory brainstem responses (ABRs) evoked by clicks **(A)** and 24 kHz tone burst stimuli **(B)**.

**Figure 5:**
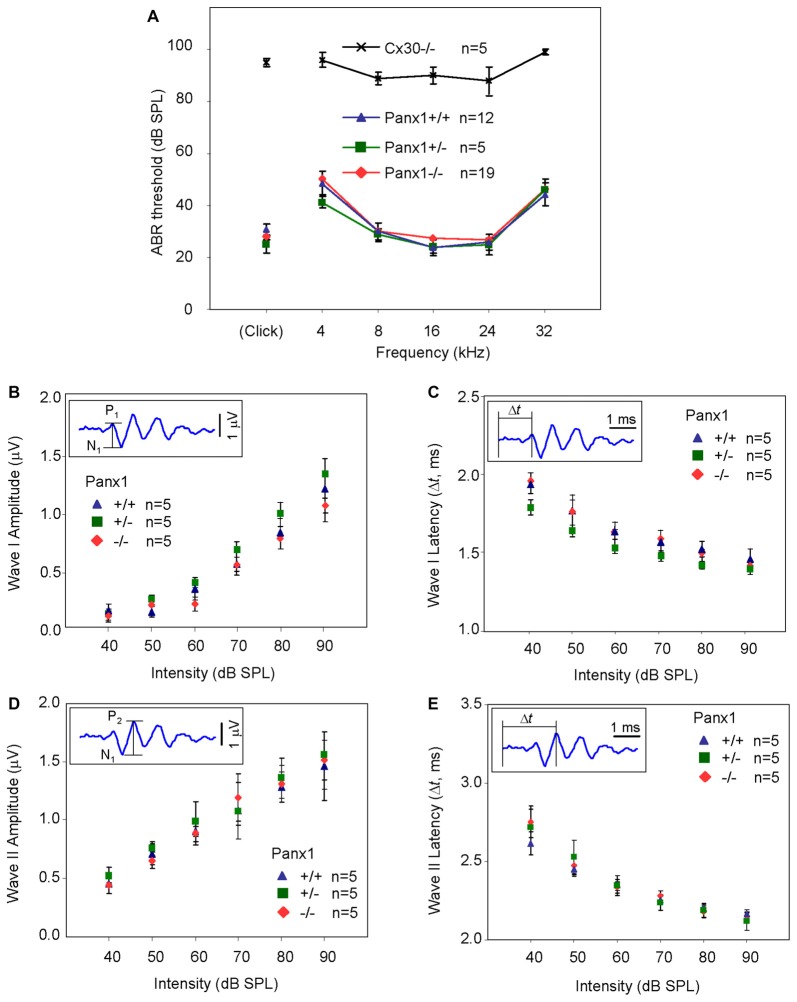
ABR analysis. **(A)** Hearing threshold estimated from Wave II for clicks and tone bursts at 4, 8, 16, 24, 32 kHz; *Cx30*−/− mice were used as an internal control. **(B)** Amplitude of Wave I (see inset) evoked by 24 kHz tone vs. stimulus intensity. **(C)** Latency of Wave I (see inset) evoked by 24 kHz tone pips vs. stimulus intensity. **(D)** Amplitude of Wave II (see inset) evoked by 24 kHz tone vs. stimulus intensity. **(E)** Latency of Wave II (see inset) evoked by 24 kHz tone pips vs. stimulus intensity. Error bars represent SEM. See also Tables 1–5.

Hearing threshold estimates from click and pure-tone ABR analysis, as well as latency and amplitude of Wave I and Wave II in *Panx1*−/− mice aged between P30 and P90, were indistinguishable from those of age-matched *Panx1*+/− and WT control mice (Figures 4, 5, Tables 1–5).

**Table 1:** Auditory brainstem response (ABR) thresholds for click stimuli and for tone bursts at 4, 8, 16, 24, 32 kHz obtained from *Panx1*+/+, *Panx1*+/− and *Panx1*−/− mice.

	*Panx1*+/+ *n* = 12	*Panx1*+/− *n* = 5	*Panx1*−/− *n* = 19
Click	31 (5)	27 (4) [*p* = 0.096]	28 (4) [*p* = 0.096]
4 kHz	48 (12)	41 (7) [*p* = 0.125]	52 (12) [*p* = 0.47]
8 kHz	30 (8)	29 (4) [*p* = 0.73]	31 (8) [*p* = 0.85]
16 kHz	24 (7)	24 (5) [*p* = 0.94]	27 (8) [*p* = 0.21]
24 kHz	26 (8)	25 (5) [*p* = 0.79]	26 (8) [*p* = 0.79]
32 kHz	44 (11)	46 (8) [*p* = 0.71]	47 (14) [*p* = 0.59]

*Shown are mean values in dB SPL, rounded to nearest integer, with standard deviation (in round brackets), and paired t-test p-values of each group relative to Panx1+/+ (i.e., WT) controls (in square brackets)*.

**Table 2:** Wave I amplitude-intensity functions across animals in response to 24 kHz tone bursts.

dB SPL	*Panx1*+/+ *n* = 5	*Panx1*+/− *n* = 5	*Panx1*−/− *n* = 5
90	1.23 (0.44)	1.08 (0.656) [*p* = 0.44]	1.37 (0.37) [*p* = 0.39]
80	0.85 (0.28)	0.82 (0.361) [*p* = 0.91]	1.02 (0.33) [*p* = 0.40]
70	1.58 (0.314)	0.58 (0.32) [*p* = 0.99]	0.72 (0.16) [*p* = 0.21]
60	0.38 (0.187)	0.24 (0.11) [*p* = 0.56]	0.44 (0.08) [*p* = 0.47]
50	0.17 (0.09)	0.25 (0.13) [*p* = 0.52]	0.29 (0.04) [*p* = 0.07]
40	0.413 (0.06)	0.11 (0.04) [*p* = 0,68]	0.16 (0.04) [*p* = 0.41]

*Shown are mean values in μV, with standard deviation (in round brackets), and paired t-test p-values of each group relative to Panx1+/+ (i.e., WT) controls (in square brackets)*.

**Table 3:** Wave I latency-intensity functions across animals in response to 24 kHz tone bursts.

dB SPL	*Panx1*+/+ *n* = 8	*Panx1*+/− *n* = 5	*Panx1*−/− *n* = 6
90	1.42 (0.17)	1.38 (0.07) [*p* = 0.64]	1.36 (0.07) [*p* = 0.52]
80	1.48 (0.15)	1.47 (0.05) [*p* = 0.89]	1.39 (0.05) [*p* = 0.24]
70	1.54 (0.16)	1.56 (0.08) [*p* = 0.77]	1.44 (0.07) [*p* = 0.627]
60	1.61 (0.16)	1.60 (0.06) [*p* = 0.92]	1.50 (0.05) [*p* = 0.19]
50	1.74 (0.25)	1.74 (0.18) [*p* = 0.98]	1.61 (0.04) [*p* = 0.28]
40	1.92 (0.25)	1.94 (0.28) [*p* = 0.92]	1.76 (0.10) [*p* = 0.21]

*Shown are mean values in ms, with standard deviation (in round brackets), and paired t-test p-values of each group relative to *Panx1*+/+ (i.e., WT) controls (in square brackets)*.

**Table 4:** Waves II amplitude-intensity functions across animals in response to 24 kHz tone bursts.

dB SPL	*Panx1*+/+ *n* = 5	*Panx1*−/− *n* = 5	*Panx1*−/− *n* = 5
90	1.43 (0.40)	1.53 (0.65) [*p* = 0.786]	1.49 (0.38) [*p* = 0.837]
80	1.26 (0.19)	1.33 (0.39) [*p* = 0.703]	1.28 (0.36) [*p* = 0.880]
70	1.05 (0.35)	1.05 (0.28) [*p* = 0.984]	1.16 (0.46) [*p* = 0.675]
60	0.87 (0.18)	0.96 (0.38) [*p* = 0.668]	0.86 (0.22) [*p* = 0.903]
50	0.68 (0.16)	0.73 (0.13) [*p* = 0.590]	0.62 (0.16) [*p* = 0.624]
40	0.42 (0.13)	0.49 (0.17) [*p* = 0.496]	0.41 (0.17) [*p* = 0.935]

*Shown are mean values in μV, with standard deviation (in round brackets), and paired t-test p-values of each group relative to *Panx1*+/+ (i.e., WT) controls (in square brackets)*.

**Table 5:** Waves II latency-intensity functions across animals in response to 24 kHz tone bursts.

dB SPL	*Panx1*+/+ *n* = 8	*Panx1*−/− *n* = 5	*Panx1*−/− *n* = 6
90	2.16 (0.08)	2.12 (0.13) [*p* = 0.55]	2.14 (0.06) [*p* = 0.60]
80	2.2 (0.07)	2.19 (0.10) [*p* = 0.84]	2.18 (0.09) [*p* = 0.66]
70	2.23 (0.09)	2.24 (0.11) [*p* = 0.86]	2.28 (0.08) [*p* = 0.67]
60	2.35 (0.10)	2.34 (0.14) [*p* = 0.89]	2.33 (0.09) [*p* = 0.70]
50	2.14 (0.9)	2.53 (0.24) [*p* = 0.27]	2.47 (0.17) [*p* = 0.33]
40	2.62 (0.21)	2.71 (0.26) [*p* = 0.53]	2.75 (0.25) [*p* = 0.32]

*Shown are mean values in ms, with standard deviation (in round brackets), and paired t-test p-values of each group relative to *Panx1*+/+ (i.e., WT) controls (in square brackets)*.

**Figure 6:**
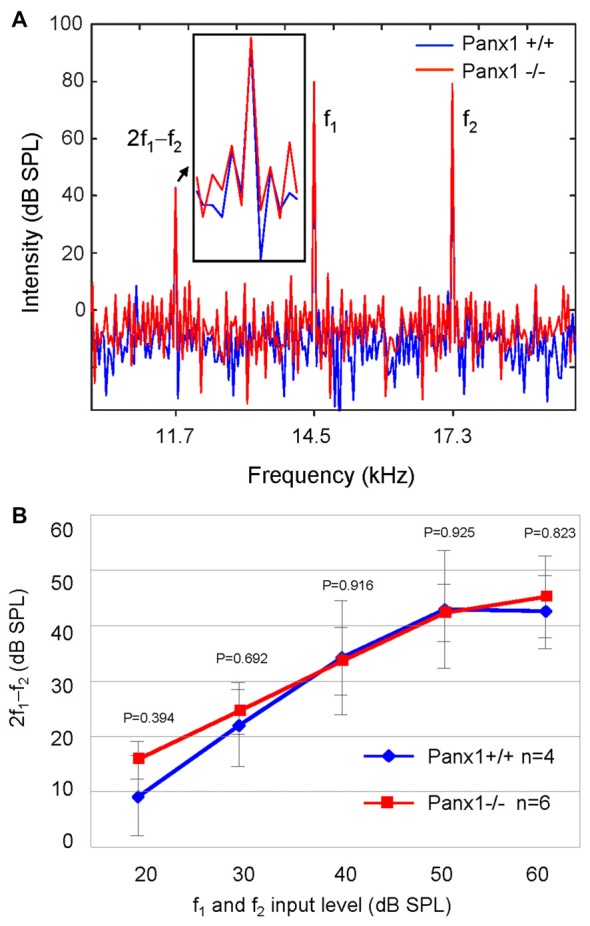
Analysis of the 2f_1_ − f_2_ cubic distortion product. **(A)** Representative spectra from WT (*Panx1*+/+, blue) and *Panx1*−/− mice (red) for a pair of 80 dB sound pressure level (SPL) primary frequencies (f_1_ and f_2_); the inset shows a magnified view of the spectrum in the region of the 2f_1_ − f_2_ cubic distortion product. **(B)** Growth function of the 2f_1_ − f_2_ cubic distortion product; error bars represent SEM; *p*-values were determined by two-tail *t*-test.

Sound generated within the mammalian inner ear as a reflection of outer hair cell (OHC) mechanical activity (Nobili and Mammano, 1996; Nobili et al., 2003) can be detected with a sensitive microphone placed in the auditory meatus (Kemp, 1978, 2002). Therefore, as a further non-invasive indicator of cochlear function, we measured the cubic (2f_1_ − f_2_) DPOAE (see “Materials and Methods” section) and found no significant differences in the DPOAE growth function of *Panx1*−/− mice and age-matched WT controls (Figure 6).

Altogether, these results indicate absence of detectable defects in auditory function of *Panx1*−/− mice. This conclusion is based on their normal hearing sensitivity, normal function of the outer hair cell-based “cochlear amplifier” (Frolenkov et al., 1998; Nobili et al., 1998; Ashmore, 2008) and absence of cochlear nerve defects.

### Connexin Expression and Function Is Normal in *Panx1*−/− Mice

Pannexins bear significant sequence homology with the invertebrate gap junction proteins, innexins, and more distant similarities in their membrane topologies and pharmacological sensitivities with the gap junction proteins, connexins (Sosinsky et al., 2011).

**Figure 7:**
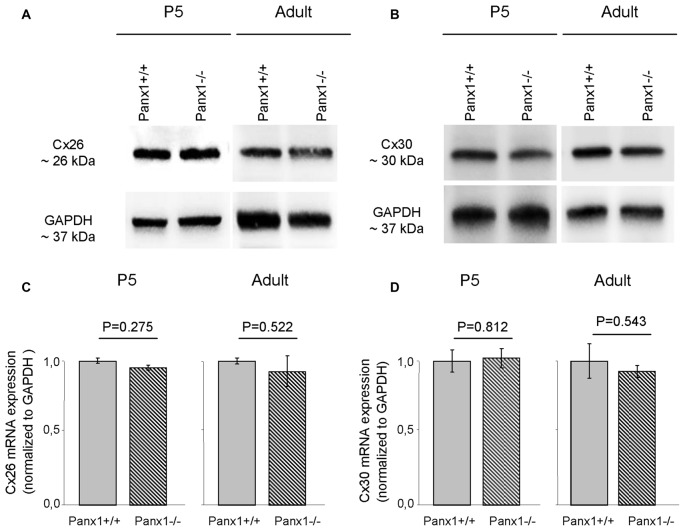
Level of Cx26 and Cx30 expression in *Panx1*−/− and *Panx1*+/+ (i.e., WT) cochleae. **(A,B)** Western blots antibodies selective for Cx26 **(A)** and Cx30 **(B)**. **(C,D)** QPCR results for mRNA transcription in P5 and adult cochleae mice from WT and *Panx1*−/− mice (*n* = 3) normalized to WT, for Cx26 **(C)** and Cx30 **(D)**. Error bars represent SEM; *p*-values were determined by two-tail *t*-test.

**Figure 8:**
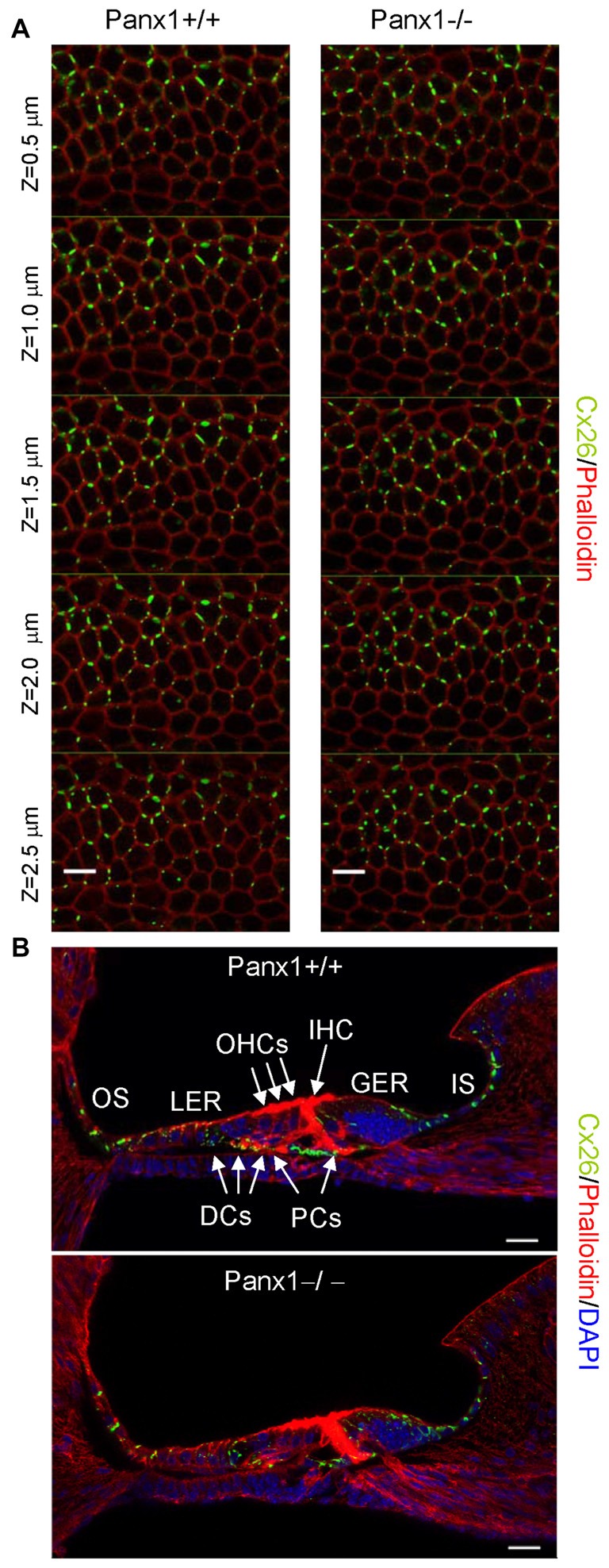
Cx26 expression pattern in the sensory epithelium of *Panx1*+/+ (i.e., WT) and *Panx1*−/− cochleae from P5 mice. **(A)** Confocal through-focus sequence (*z*-stack) acquired at 0.5 μm increments showing Cx26 expression detected with a selective antibody (green) in epithelial cells of the LER counterstained with phalloidin (red); scale bars: 10 μm.** (B)** Representative transversal sections of the organ of Corti. Shown are maximal projection rendering of three consecutive midmodiolar confocal optical sections taken at 0.5 μm intervals in the medial cochlear turn; nuclei were stained with DAPI (blue). IHC, inner hair cells; OHCs, outer hair cells; DCs, Deiters’ cells; LER, lesser epithelial ridge; greater epithelial ridge (GER) greater epithelial ridge; PCs, Pillar cells; scale bars: 20 μm.

Non-sensory cells of the mammalian cochlea express two closely related gap junction proteins, connexin 26 (Cx26) and connexin 30 (Cx30; Lautermann et al., 1998; Ahmad et al., 2003; Forge et al., 2003; Zhao et al., 2006), the expression of which is coordinately regulated (Ortolano et al., 2008). Mouse models indicate that altered expression levels of these connexins in the early postnatal days impacts on organ of Corti development and hair cell maturation (Johnson et al., 2017), preventing normal hearing acquisition (Cohen-Salmon et al., 2002; Teubner et al., 2003; Ahmad et al., 2007; Sun et al., 2009; Crispino et al., 2011, 2017; Qu et al., 2012; Zhu et al., 2013). As regulatory mechanism may potentially be shared between connexins and pannexins, we examined the expression of Cx26 and Cx30 by Western blot analysis and QPCR, and found no significant differences between *Panx1*−/− mice and age-matched WT controls (Figure 7). The spatial distribution of Cx26 at P5 was investigated also by immunofluorescence and, again, no differences between *Panx1*−/− mice and age-matched WT controls were detected (Figure 8).

**Figure 9:**
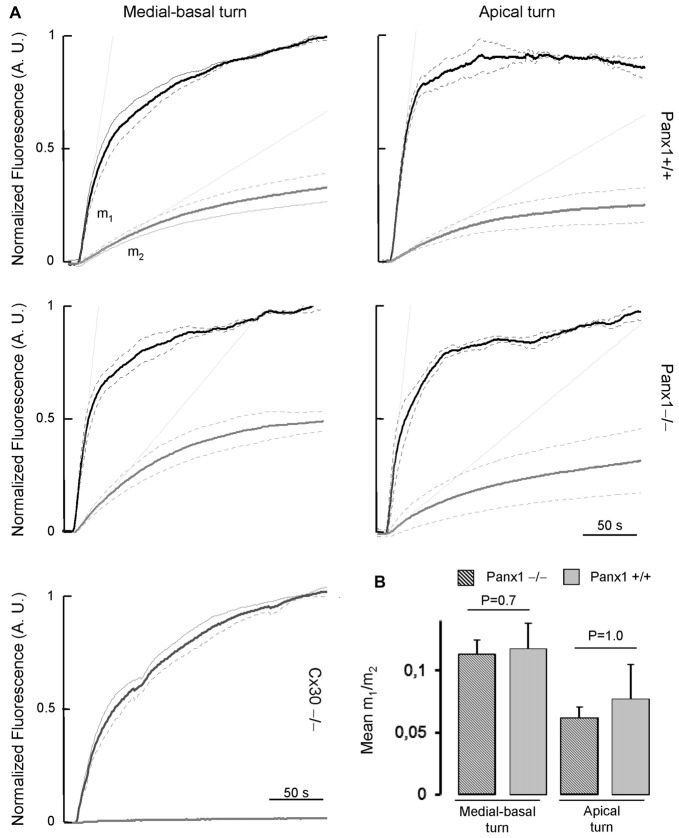
**(A)** Lucifer yellow fluorescence emission averaged over the cell body of (first order) cells (gray solid lines, *n* = 5) adjacent to the injected cell and normalized to the maximal fluorescence emission detected in the injected cell (black solid lines); data are mean values ± SEM (dot lines) for *n* = 8 cells in each condition. **(B)** For each experiment, the interpolating line of the curve related to first order cells (with computed slope *m*_2_) and to the injected cell (with computed slope *m*_1_) was computed over the first 10 s of recording. Histograms show mean values of the ratio between *m*_2_ and *m*_1_ for WT (*Panx*+/+, dashed bars) and *Panx1*−/− mice (filled bars). Error bars represent SEM.

To assess whether the expressed connexins confer cell-to-cell connectivity, we quantified dye transfer in the LER of organotypic cochlear cultures from P5 mice (see “Materials and Methods” section). To gauge transfer rate, we measured the slope of the LY fluorescence growth function at the onset of dye delivery in the donor cell (*m*_1_) and in its nearest neighbors (*m*_2_), and found no significant differences in the *m*_2_/*m*_1_ ratio of *Panx1*−/− mice and WT controls (Figure 9).

Altogether the results presented in Figures 7–9 indicate that lack of Panx1 in *Panx1*−/− mice impacts neither on connexins expression, nor on gap-junction coupling in the developing organ of Corti.

### Spontaneous Ca^2+^ Signaling Activity Is Normal in the Developing Cochlea of *Panx1*−/− Mice

Connexins play a crucial development role in the postnatal cochlea (as mentioned above), also by supporting ICS activity both in the LER (Beltramello et al., 2005; Piazza et al., 2007; Anselmi et al., 2008; Majumder et al., 2010; Ceriani et al., 2016) and in the greater epithelial ridge (GER; Tritsch et al., 2007; Schütz et al., 2010; Rodriguez et al., 2012; Wang and Bergles, 2015; Johnson et al., 2017; Mammano and Bortolozzi, 2017). Using focal ATP delivery or photostimulation with caged IP_3_, Anselmi et al. (2008) showed ICS failure in cultures with deficient expression of Cx26 and Cx30, whereas ICS in organotypic cultures from *Panx1*−/− mice was indistinguishable from those of WT controls.

**Figure 10:**
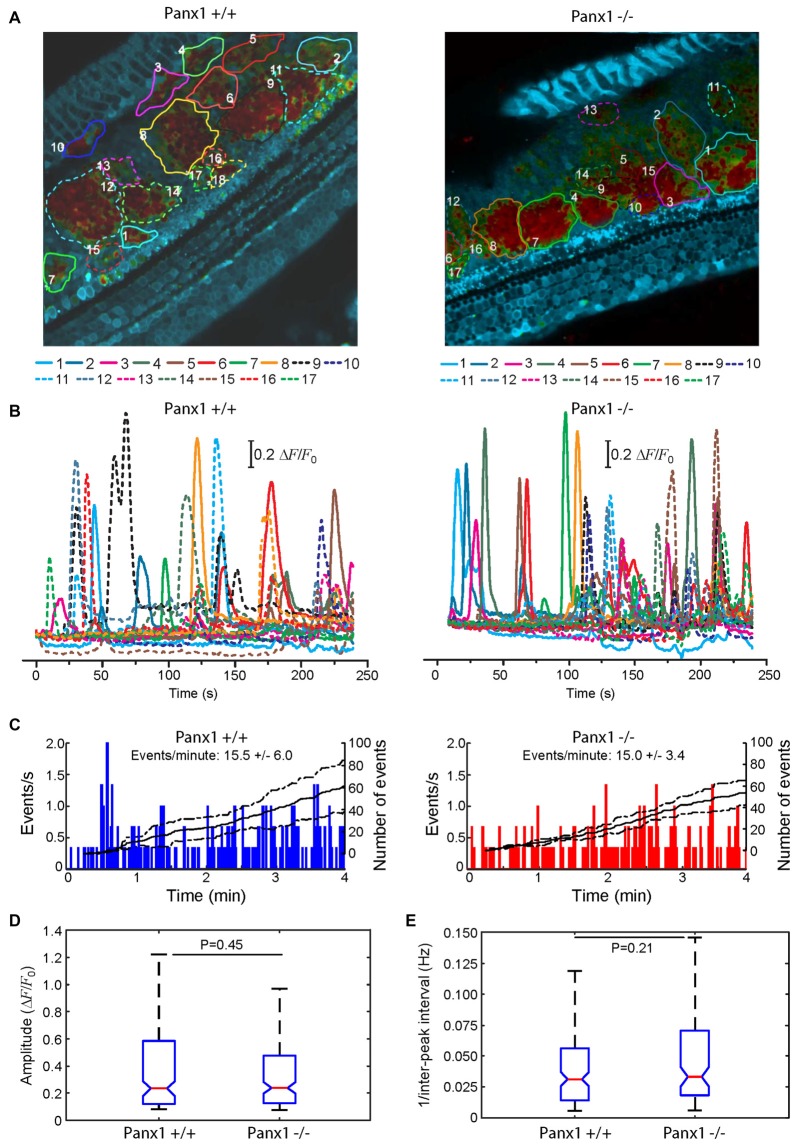
Spontaneous cytosolic Ca^2+^ transients in the GER of WT (*Panx*+/+) and *Panx1*−/− postnatal cochlear cultures. **(A)** Representative false-color images of Fluo-Forte fluorescence changes (Δ*F*/*F*_0_), encoded as shown by the color scale bar, obtained as maximal projection rendering of all frames recorded in an apical-middle turn culture from a P5 mouse imaged for 4 min at 5 frames per second; regions of interests are shown superimposed on different GER areas invaded by spontaneous Ca^2+^ waves. Scale bar, 50 μm. **(B)** Fluo-forte traces generated as pixel averages from the color-matched regions of interests shown in **(A)**. **(C)** Frequency histograms of spontaneous cytosolic Ca^2+^ transients (events) in cultures from P5 *Panx*+/+ and *Panx1*−/− mice (pooled data). **(D)** Distributions of events amplitude (Δ*F*_max_/*F*_0_), from data in **(C)**. The values of the median and inter-quartile range (IQR) are, respectively: 0.2340 and 0.4641 (*Panx*+/+); 0.2398 and 0.3501 (*Panx1*−/−). **(E)** Distributions of the frequency (i.e., the reciprocal of the inter-peak interval) between consecutive events from data in **(C)**. The values of the median and IQR are, respectively (in Hz): 0.0312 and 0.0420 (Panx+/+); 0.0331 and 0.0520 (*Panx1*−/−). *P*-values in **(C,D)** were computed with the Mann-Whitney *U* test.

Here, we used multiphoton microscopy to monitor spontaneous ICS activity (Tritsch et al., 2007; Schütz et al., 2010; Rodriguez et al., 2012; Wang and Bergles, 2015; Johnson et al., 2017; Mammano and Bortolozzi, 2017) in organotypic cochlear cultures from P5 mice loaded with the Ca^2+^ indicator Fluo Forte AM (Mammano and Bortolozzi, 2017). Specifically, we examined the frequency of occurrence of spontaneous Ca^2+^ transients (events) in the apical cochlear turn by counting all occurrences within the portion of the GER in the field view from P5 Panx1−/− mice and age–matched WT siblings (Figure 10). We found a similar mean frequencies of occurrence (15.5 ± 6.0 events/min in Panx1+/+ vs. 15.0 ± 3.4 events/min in Panx1−/− cultures). Likewise, amplitude and inter-peak interval distributions of spontaneous Ca^2+^ transients of Panx1−/− cultures overlapped with those of Panx1+/+ cultures. Altogether, these results indicate that spontaneous ICS activity in the GER of the postnatal cochlea is not affected by Panx1 ablation.

## Discussion

The *Panx1*−/− strain we have analyzed was the first with a reported global ablation of *Panx1* (Anselmi et al., 2008; Bargiotas et al., 2011). The present results confirm successful ablation of *Panx1* in *Panx1*−/− mice, while our ABR and DPOAE data indicate normal hearing sensitivity, normal function of the outer hair cell-based “cochlear amplifier” (Frolenkov et al., 1998; Nobili et al., 1998; Ashmore, 2008) and absence of cochlear nerve defects, in agreement with the initial observation that *Panx1*−/− mice do not exhibit a detectable hearing phenotype (Anselmi et al., 2008,^8^).

We also confirmed that lack of *Panx1* affects neither the expression of inner ear connexins nor gap junction communication in the organ of Corti. Furthermore, our experiments with cochlear organotypic cultures indicate that the ATP-release mechanism underlying the spontaneous ICS activity of cells in the GER is intact. Preservation of this mechanism is essential for hearing acquisition (Schütz et al., 2010; Rodriguez et al., 2012; Mammano and Bortolozzi, 2017) and maturation of sensory hair cells (Johnson et al., 2017). Recent results with a monoclonal antibody that inhibits Cx26 hemichannels substantiate the notion that, in the cochlear sensory epithelium, ATP is released from such hemichannels (Xu et al., 2017).

Ensuring that an allele derived from the tm1a cassette is a full null, rather that a hypomorph such as the *Panx1^tm1a(KOMP)Wtsi^* strain, and alleviating potential off-target gene mis-regulation, requires modification of tm1a, which can be performed in embryonic stem (ES) cells or in crosses with transgenic Flp and Cre mice. Flp deletion converts tm1a to a conditional allele (tm1c), restoring gene activity, whereas the promoter-driven selection cassette and floxed exon of the tm1a allele can be deleted by Cre to generate a lacZ-tagged allele (tm1b; Skarnes et al., 2011). This is usually accomplished by breeding the mice to a source of Cre expressed in the germline, followed by outcrossing and selection of knockout offspring that fail to carry the Cre driver (Skarnes et al., 2011). This was also the strategy followed by the International Mouse Phenotyping Consortium (IMPC^9^ Brown and Moore, 2012) to convert the tm1a allele to tm1b for subsequent phenotyping (Ryder et al., 2014). The *Panx1*^tm1b(KOMP)Wtsi^ strain generated in this way was analyzed by the IMPC and reported to have no significant hearing/vestibular phenotype^10^.

Thus, altogether, our results and conclusions are consistent with hearing assessments both in the Genentech-*Panx1*−/− strain (Abitbol et al., 2016) and in the *Panx1*^tm1b(KOMP)Wtsi^ strain generated and analyzed by the IMPC.

The lack of any measurable auditory phenotype in three strains, which are all global knockouts of *Panx1*, is in stark contrast with the phenotype reported for *Pax2-cPanx1−/−* (Zhao et al., 2015), and even more so for *Foxg1*-*cPanx1*−/− mice that, despite descending from the hypomorphic *Panx1*^tm1a(KOMP)Wtsi^ strain, retained strong immunoreactivity for the Panx1 #4515 chicken anti-human antibody (which is not specific: Bargiotas et al., 2011; see “Introduction” section) in the organ of Corti and the spiral limbus (Chen et al., 2015). However, it should be considered that both the *Pax2-cPanx1−/−* and the *Foxg1*-*cPanx1*−/− strain must necessarily express a Cre recombinase (Friedel et al., 2011), whereas the three global knockout strains mentioned above do not express Cre. It is well known that Cre expression in mammalian cells can induce chromosomal aberrations and toxicity that is dependent on the level of Cre activity (Loonstra et al., 2001). Indeed, Caspase-3 activation and cell degeneration are reported hallmarks of the organ of Corti in *Pax2-cPanx1*−/− mice (Zhao et al., 2015). Furthermore, *Foxg1*^Cre^ mice that are homozygous for the targeted mutation die perinatally (Tian et al., 2012,^11^), whereas heterozygous *Gfi1*^Cre^ mice, one of the many driver lines used for conditional cell-specific gene deletion/reporter gene activation in the inner ear (Cox et al., 2012), were recently reported with an early onset progressive hearing loss, which was absent in their wild-type littermates (Matern et al., 2017).

Another risk factor that is worth considering is related to the structure of the tm1a selection cassette (Testa et al., 2004; Skarnes et al., 2011) used to generate the hypomorphic *Panx1^tm1a(KOMP)Wtsi^* mice. As the latter were not crossed with a Flp deleter line before being used to create *Pax2-cPanx1−/−* and *Foxg1*-*cPanx1*−/− mice, it is not clear from the data provided if the Cre-mediated deletion removed only exon 2 of *Panx1* or also the *neo* cassette, nor from which tissue the sample tested was obtained.

It is well known that retention of a *neo* cassette can cause unexpected phenotypes in “knockout” mice due to neighborhood effects (Pham et al., 1996; Scacheri et al., 2001; Ren et al., 2002; Meier et al., 2010). Indeed, removal of the *neo* cassette and critical exon from the tm1a allele is regarded as an essential procedure that alleviates potential off-target gene mis-regulation caused by the *neo* promoter, and to ensure that the allele is a full null rather then a hypomorph (Skarnes et al., 2011).

In this vein, it was recently argued that the hearing loss phenotype exhibited by *Cx30*−/− mice (Teubner et al., 2003) depends on the cumulative effect of deletion of *Cx30* and 3’ insertion of a *lacZ* and *neo* cassette. Indeed, in a strictly related knockout mouse model (*Cx30*Δ/Δ) in which *Cx30* was removed without perturbing the surrounding sequences, hearing thresholds determined by ABR analysis are normal (Boulay et al., 2013; Crispino et al., 2017).

In conclusion, our extended characterization of *Panx1−/−* mice provides strong evidence that *Panx1* is dispensable for hearing acquisition and auditory function.

## Data and Code Availability

Data and computer code used to analyze data are available from the authors upon request.

## Author Contributions

FaM designed the studies, provided resources to conduct the studies and wrote the manuscript; HM provided Panx1−/− mice and genotyping protocols; VZ, MP and FC performed animal genotyping; MR and FS were in charge of animal welfare and performed quality controls; VZ and FP performed *in vivo* electrophysiology; AC wrote software code to filter ABR waveforms; CDC wrote image acquisition and analysis software; VZ, FP and CN analyzed ABR and DPOAE data; VZ performed immunofluorescence studies; FP performed Western blot analyses; VZ and GZ generated organotypic cochlear cultures; GZ performed patch clamp, dye transfer in cochlear organotypic cultures and analyzed data; CP and FlM performed multiphoton microscopy and Ca^2+^ imaging in cochlear organotypic cultures, and analyzed data; AMS, ARF and FaM supervised the work of junior colleagues; VZ, GC, FC, FS, ARF and AMS edited the text.

## Conflict of Interest Statement

The authors declare that the research was conducted in the absence of any commercial or financial relationships that could be construed as a potential conflict of interest.

## Footnotes

^1^http://www.genenames.org/cgi-bin/genefamilies/set/288

^2^http://www.informatics.jax.org/allele/MGI:5310802

^3^http://www.informatics.jax.org/marker/phenotypes/MGI:1860055

^4^http://www.knockoutmouse.org

^5^http://www.eucomm.org

^6^http://www.komp.org/alleles.php#conditional-promoter-csd

^7^https://www.infrafrontier.eu/

^8^http://www.informatics.jax.org/marker/phenotypes/MGI:1860055

^9^http://www.mousephenotype.org/

^10^www.mousephenotype.org/data/genes/MGI:1860055#section-associations

^11^http://jaxmice.jax.org/strain/004337.html
